# Digital versus paper-based voiding diaries: a feasibility study of usability, data quality, patient satisfaction and adherence

**DOI:** 10.1007/s00345-026-06609-5

**Published:** 2026-07-20

**Authors:** M. J. Wenk, L. V. Renner, J. Lockl, P. Fechner, N. Ruhland, N. Carl, M. H. Mangold, H. Krause, N. Westhoff

**Affiliations:** 1 Department of Urology and Urological Surgery, University Medical Centre Mannheim, University of Heidelberg, Theodor-Kutzer-Ufer 1-3, 68167 Mannheim, Germany; 2 University Medical Centre Mannheim, Theodor-Kutzer-Ufer 1-3, 68167 Mannheim, Germany; 3 InContAlert GmbH, Erlanger Str. 21, 95444 Bayreuth, Germany

**Keywords:** Electronic voiding diary, Paper-based voiding diary, System usability scale, Digital health, Postoperative continence, eHealth

## Abstract

**Purpose:**

This study aims to compare the effectiveness, user experience, usability, and clinical utility of an electronic voiding diary (EVD) versus a traditional paper-based voiding diary (PVD) in patients undergoing robot-assisted radical prostatectomy (RARP).

**Methods:**

In this exploratory feasibility study, 29 patients completed both an EVD and PVD following catheter removal, documenting micturition, pad use, and fluid intake. Usability was assessed using the System Usability Scale (SUS), task performance, and questionnaires on digital habits. Data were stratified by age, screen time, and app usage.

**Results:**

This study included 29 men (mean age 65.24 ± 6.73 years) after RARP. SUS scores were higher in patients with regular app use (79.85 vs. 43.33; *p* = 0.024). In patients with ≥ 2 h daily screen time SUS Scores were 80.42 vs. 59.32 (< 2 h daily screen time; *p* = 0.139). Regular app users consistently preferred the EVD across all four evaluated categories: usability (regular app use 1.19 EVD vs. infrequent app use 2.17 PVD; *p* = 0.014), misunderstandings (0.25 PVD vs. 1.83 EVD; *p* = 0.049), learnability (1.00 EVD vs. 2.33 PVD; *p* = 0.030), and graphical presentation (3.38 EVD vs. 0.33 EVD; *p* = 0.020).

**Conclusion:**

This feasibility study demonstrates that EVDs are practical, reliable, and well-accepted for early postoperative monitoring after RARP. Usability was strongly influenced by age and digital experience, with younger, regular app users with higher daily screen time performing better. These findings support further development and broader implementation of EVDs in urological care.

**Supplementary Information:**

The online version contains supplementary material available at 10.1007/s00345-026-06609-5.

## Introduction

In surgical urology, close monitoring of bladder function is crucial after procedures like prostatectomy or cystectomy. These surgeries can impair continence, increase residual urine volumes, and reduce bladder storage capacity [1, 2]. Documenting postoperative voiding patterns helps to identify potential complications early and supports individualized postoperative treatment decisions [3]. Beyond their role in the postoperative setting, voiding diaries are also commonly used to assess voiding dysfunction [4]. They provide valuable insights into bladder habits and symptoms across many patient populations [5].

Traditionally, these diaries have been recorded on paper, where patients manually document their voiding patterns, fluid intake, pad use and associated symptoms. While paper-based voiding diaries (PVDs) are widely used due to their simplicity and low cost, they are often associated with issues such as incomplete entries, patient recall bias, and non-compliance [6].

In recent years, the development of electronic voiding diaries (EVDs) has offered a promising alternative. EVDs aim to enhance data accuracy, improve patient engagement, and reduce the burden of documentation by leveraging real-time input [7]. Beyond ecological benefits, the EVD is expected to improve patient experience with a clearer and more intuitive data entry process. For healthcare providers, it should enable faster access to patient data and support clinical decision-making [8]. Given the critical impact on patient adherence and data reliability, thorough usability testing is imperative prior to the large-scale implementation of eHealth solutions [9].

This study aims to compare the effectiveness, user experience, usability, and clinical utility of an electronic versus a paper-based voiding diary A digital, app-based EVD has been developed and specifically customised for urological practice which was tested in a cohort of patients undergoing robot-assisted radical prostatectomy (RARP).

## Patients and methods

### Patient enrollment

After institutional ethics review board approval (Nr. 2022 − 641) was received, a cross-sectional explorative feasibility cohort study was performed. Patient data were collected by soliciting volunteers who underwent RARP for localized prostate cancer with curative intent at our tertiary care center from May 7th, 2024 to September 3rd, 2024 upon consenting. Recruitment was restricted to a distinct urology ward with specialized technological equipment, where each bed was equipped with an individual monitor, as required for app use. All eligible RARP patients on that ward were approached; three declined and the remaining patients consented (*n* = 29). Out of these, 24 patients completed the questionnaire validly and were included in the final analysis.

### Clinical parameters

Patient characteristics and clinical data were collected retrospectively from medical records. The variables included age, date of surgery and tumor stage. Additionally, the number of days until catheter removal and the results of micturition cystourethrography (MCU), when applicable, were recorded.

### Introduction of PVD and EVD

The urinary catheter was removed on postoperative day four, with or without a prior MCU, determined at the surgeon´s discretion. Following catheter removal, patients were given a PVD and instructed in its use. At the same time, they were given their individual login credentials for their EVD accounts. Upon first login, the app guided users through its functions via integrated instructional pages (Supplemental Fig. 1).

Patients then used both PVD and EVD to record real-time void volumes, measured with a standardized cup. Pad usage, indicating early post-operative incontinence, was also documented. The EVD was accessed via a bedside monitor that was integrated into each hospital bed individually. Data entries were automatically synchronized with the hospital’s internal system at 24-hour intervals.

The app was developed by *inContAlert GmbH*, which also ensured secure data transmission and maintained the necessary interfaces for continuous, real-time integration with hospital IT systems. The user interface was co-designed with the clinical team to optimize usability and meet data protection standards.

### Questionnaire

Participants completed two questionnaires, which assessed their daily technological habits and their comparative evaluation of the EVD versus PVD.

The System Usability Scale (SUS; Brooke, 1996) is a widely used and well-validated instrument for assessing the usability of digital health applications [10]. It consists of 10 items; each rated on a 5-point Likert scale ranging from 1 (“strongly disagree”) to 5 (“strongly agree”). The items alternate between positive and negative formulations to prevent response bias.

To calculate the total SUS score, responses are first re-coded: for each odd-numbered item, 1 is subtracted from the participant’s rating; for each even-numbered item, the rating is subtracted from 5. The resulting values are then summed across all ten items. This value is multiplied by 2.5 to derive the final SUS score, which ranges from 0 to 100, with higher scores indicating higher perceived usability [10].

The SUS has been translated into German, and its validity has been confirmed [11]. As previous studies have noted, the SUS scores may be subject to systematic positive bias, often clustering above 50 even for suboptimal systems [12]. Therefore, the present study included additional objective usability measures. Task completion time and task completion rate as complementary performance-based metrics were recorded to provide a more comprehensive evaluation of usability [13]. These performance-based metrics serve as countermeasures against the bias inherent in SUS ratings, as they provide direct, observable evidence of system efficiency and effectiveness rather than relying solely on subjective impressions.

In addition to the SUS, participants completed a self-administered questionnaire designed to collect demographic, health-related, and technology usage data. This instrument comprised 15 items, including questions on prior use of voiding diaries, familiarity with health-related apps, and general app usage habits. Furthermore, participants provided open-ended feedback regarding any difficulties encountered during the use of the application, features they particularly appreciated, and suggestions for improvement. Finally, participants completed a comparative questionnaire evaluating the EVD versus the PVD across four dimensions: perceived ease of use, clarity, learnability, and the perceived effectiveness of the graphical presentation of results. Responses were recorded on two unipolar scales (0–4) for EVD and PVD (Supplemental Materials).

Entries recorded in the voiding diaries were not analyzed statistically; comparisons between PVD and EVD were based on questionnaire-derived outcomes (e.g., usability ratings).

### Statistical analysis

For analysis, data were stratified by age (< 65 years vs. ≥65 years), as well as by self-reported regular app usage (no, yes) and daily screen time on mobile devices in general (< 2 h vs. ≥2 h per day). We selected mobile device screen time specifically because it reflects participants’ direct interaction with on-screen keyboards and mobile user interfaces. SUS scores were analyzed using between-group comparisons across predefined usage groups. No correlation or association analyses were performed.

Quantitative data are presented as mean with standard deviation (SD), and categorical data as absolute and relative frequencies. All statistical analyses, including distribution tests and Mann–Whitney U tests, were performed using RStudio (version 2025.09.2 + 418). The level of statistical significance was set at 0.05.

## Results

A total of 29 male patients with a mean age of 65.24 ± 6.73 years were included in this study. Of the patients included in the study, 2 (6.9%) were classified as having low-risk tumors, 20 (69.0%) were classified as having intermediate-risk tumors and 7 (24.1%) as high-risk, based on Union for International Cancer Control (UICC) staging criteria [14]. Postoperatively, all patients received a transurethral catheter for a standardized duration of four days.

### System usability and patient characteristics

Out of 29 patients 24 patients completed the questionnaire adequately. The SUS scores were numerically slightly higher among younger patients, although not statistically significant (< 65 years: SUS 71.46 ± 25.71 vs. ≥65 years: SUS 69.09 ± 27.37; p 0.430). Patients who reported more than 2 h of daily screen time showed a usability of the EVD of 59.32 ± 32.77 compared to 80.42 ± 12.37 in those with less screen time (p 0.139). Regular app use was associated with significantly higher SUS scores (regular users: SUS 79.85 ± 13.71 vs. infrequent users: SUS 43.33 ± 34.39; p 0.024). SUS scores are shown in Table 1. Regular app use was reported by 83% of participants younger than 65 years and by 58% of those aged 65 years or older, with no statistically significant difference between the age groups (p 0.200).

**Table 1:**
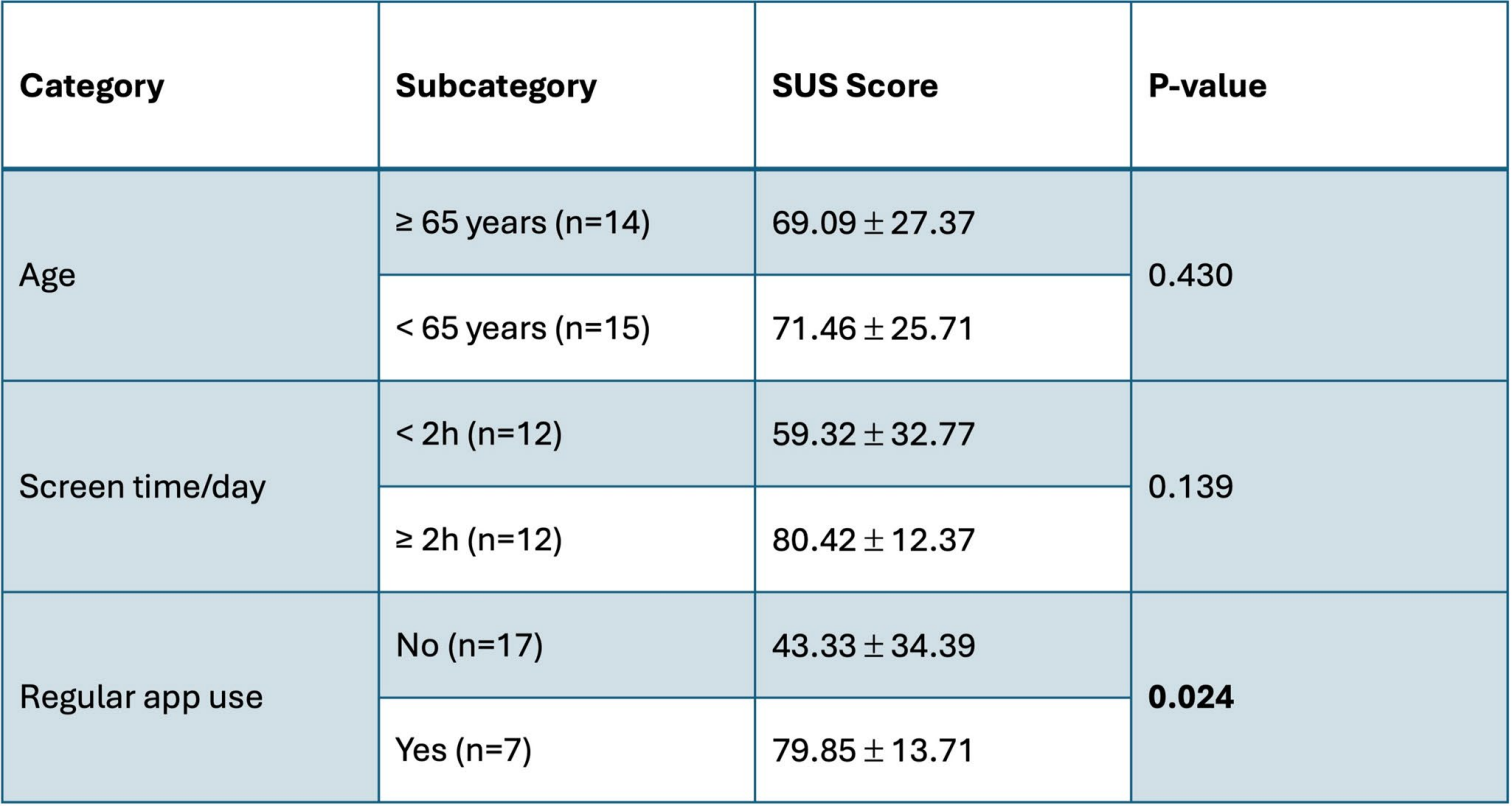
SUS scores—comparison of SUS scores across user subgroups. categories (age, screen time/day, regular app use) and their respective subgroups are shown. The mean SUS scores are displayed with standard deviations

### Task performance

The evaluation of task performance revealed an overall success rate of 92.42% across all interaction types. When evaluated by task category, the success rates were similar: micturition entries were successfully completed in 92.66% of cases, pad entries in 91.74%, and drink logging in 93.10% of attempts.

Task duration analysis showed that patients were generally able to complete entries efficiently. The median completion time for micturition tasks was 26.06 ± 24.19 s, while pad entries had a median duration of 11.11 ± 8.89 s. Drink entries took a median of 19.41 ± 24.09 s. When considering all task types combined, the median task duration was 18.34 ± 21.92 s.

### Comparison of EVD vs. PVD

Overall, the EVD was rated superior in terms of “graphical presentation” (2.61 EVD, p 0.00003). Ease of use showed a rating of 0.27 EVD (p 0.658). Further evaluation showed instances of misunderstanding with the EVD (0.30 EVD, p 0.423). The assessment of learnability showed a value of 0.04 PVD (p 0.949).

**Fig. 1:**
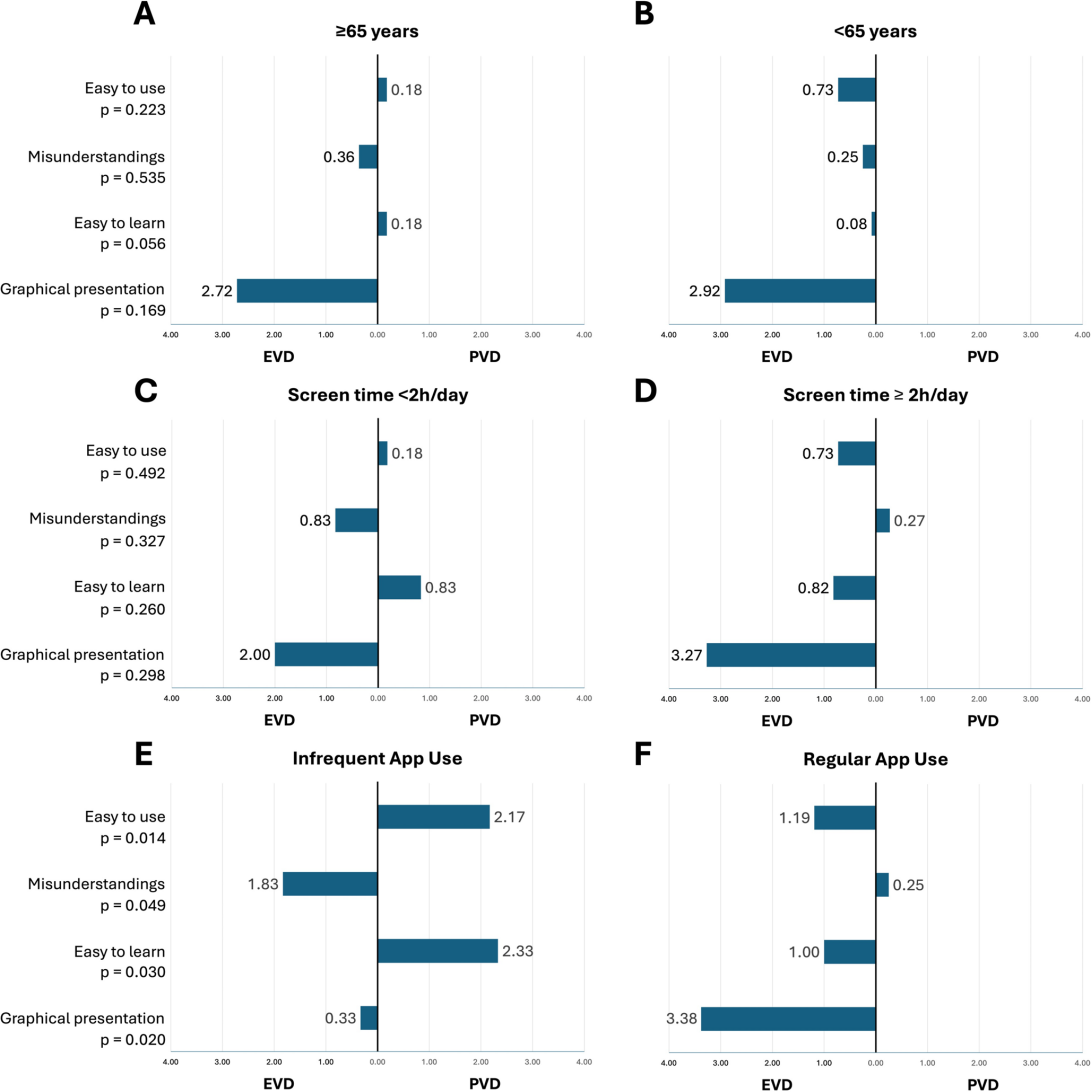
Comparison EVD vs. PVD—mean ratings on two unipolar scales (0–4) for EVD and PVD across six subgroups. Each bar represents the average response for the four dimensions: ease of use, misunderstandings, ease of learning, and graphical presentation. **A** Patients ≥ 65 years **B** Patients < 65 years **C** <2 h screen time per day **D** ≥2 h screen time per day **E** infrequent app use **F** regular app use

In the age-stratified analysis, patients younger than 65 years showed a usability value of 0.73 EVD, while patients aged 65 years or older showed a usability value of 0.18 PVD (p 0.223). The graphical presentation of the EVD received positive ratings in both age groups, with values of 2.92 EVD in patients younger than 65 years and 2.27 EVD in patients aged 65 years or older (p 0.169) (Fig. 1).

Among patients with daily screen time of at least 2 h, the learnability score was 0.82 EVD, and the usability score was 0.73 EVD. Among patients with less than 2 h of daily screen time, the learnability score was 0.83 PVD, and the usability score was 0.18 PVD (*p* = 0.260 for learnability, *p* = 0.492 for usability) (Fig. 1).

Significant differences emerged between patients with varying levels of app usage. Regular app users consistently preferred the EVD across all four evaluated categories (usability (regular app use 1.19 EVD vs. infrequent app use 2.17 PVD; p 0.014), misunderstandings (regular app use 0.25 PVD vs. infrequent app use 1.83 EVD; p 0.049), learnability (regular app use 1.00 EVD vs. infrequent app use 2.33 PVD; p 0.030), and graphical presentation (regular app use 3.38 EVD vs. infrequent app use 0.33 EVD; p 0.020)), whereas patients with infrequent app use favored the PVD in usability, learnability and misunderstandings (Fig. 1).

### Patient’s perception of the EVD

**Fig. 2:**
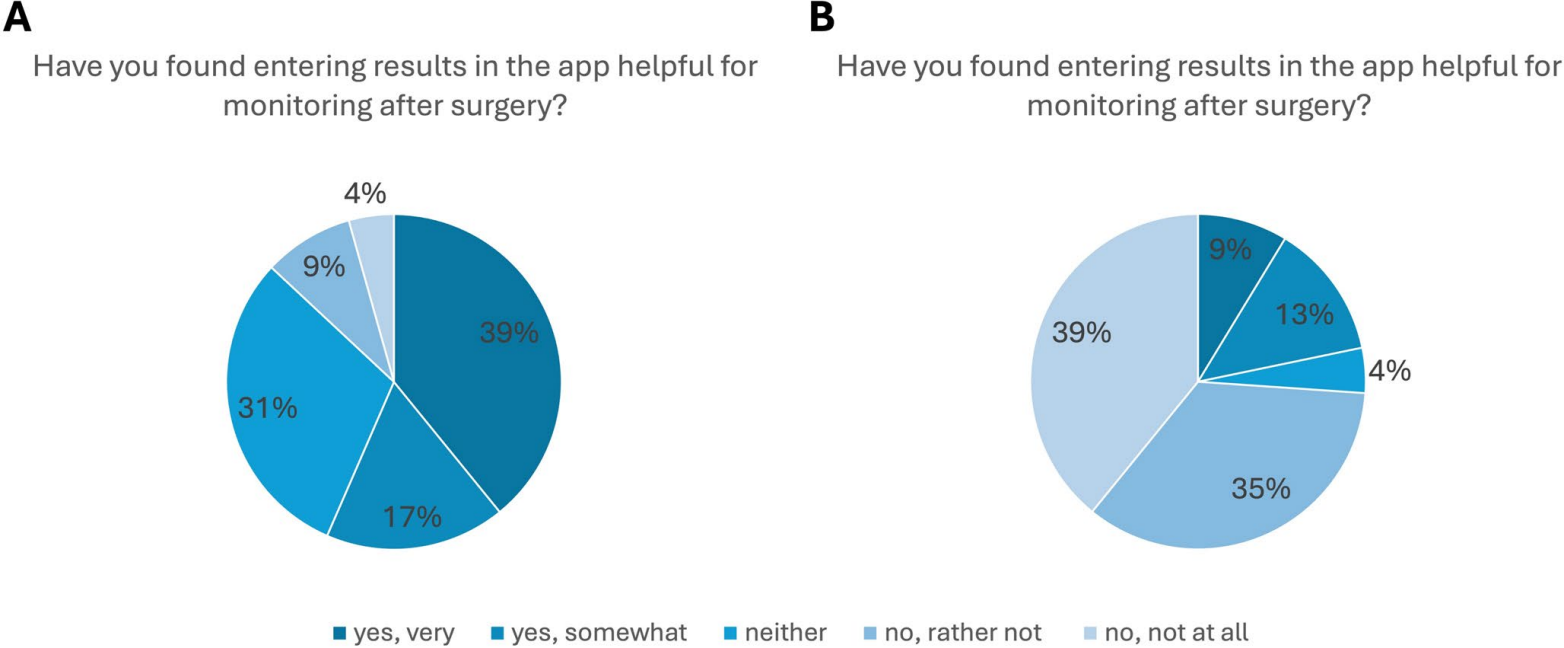
Patient perception of the EVD

More than half of the participants found the EVD helpful (39% very helpful, 17% somewhat helpful). Moreover, most patients did not perceive the use of the EVD as burdensome, with 39% reporting no burden at all and 35% reporting only minimal discomfort. When discomfort was reported, it was mostly put down to the design of the user interface (Fig. 2).

## Discussion

This feasibility study provides compelling evidence that an EVD is a practical, reliable, and user-friendly alternative to traditional PVDs in the early postoperative setting after RARP. With an overall task success rate of 92.42%, and consistently high success rates across all task categories, our findings confirm the technical stability of the digital system and the ability of patients to engage with it effectively in a real-world clinical environment.

The task duration data further support these findings. Despite the physical and cognitive challenges often encountered in the immediate postoperative period [15], patients demonstrated efficient use of the EVD, with median completion times well below half a minute across all entry types, which the hospital staff considered as an efficient completion rate. However, the relatively high variability in task completion times indicates that some patients required considerably longer for certain entries. The high variability suggests that while most patients navigated the application efficiently, roughly one in ten required substantially more time, highlighting the need for thorough onboarding and individualized support to ensure consistent usability across all user groups, which has also been discussed in prior studies by Pauls et al. and Dequirez et al. [16, 17].

These data reinforce previous reports suggesting that EVDs — when properly implemented —can significantly enhance compliance, streamline data collection, and reduce the clinician’s administrative workload [18]. Mangera et al. showed that the time to analyze paper diaries was 66% longer compared to electronic diaries and prone to interpretation errors [8]. In our setting, the automatic synchronization of diary entries with the hospital IT system enables clinicians to review patient data before morning rounds, allowing for immediate, data-driven treatment adjustments, and more personalized patient care in the future — an operational benefit emphasized in recent digital health literature [19, 20].

In line with prior research, user characteristics were a major determinant of usability. SUS scores and qualitative feedback suggested numerically higher usability ratings for the EVD among younger patients and those with greater digital exposure; however, these differences did not reach statistical significance. Participants reporting regular app usage rated the EVD significantly higher in terms of usability, no misunderstandings, learnability, and graphical presentation. This aligns with findings from Mateu Arrom et al. and Dequirez et al., who observed greater acceptance and preference for electronic diaries among younger and digitally savvy users [17, 21]. Mateu Arrom et al. provide an explanation to those age-related differences, since they found that younger patients were more likely to at all own a smartphone [22].

Regular app users not only completed tasks faster but also reported fewer misunderstandings, suggesting that prior experience with similar interfaces contributes to more efficient and confident use. In contrast, infrequent app users and older participants (≥ 65 years) showed a tendency to prefer the PVD and reported more difficulties navigating the EVD interface; however, these observations should be interpreted with caution given the small cohort size. These findings highlight the need for targeted onboarding and possibly an interface simplification for certain user groups. As noted by Dequirez et al., integrating patient education and support mechanisms is essential to ensure equitable use of digital tools across diverse populations [17].

Despite its promising usability, the EVD has limitations. While the interface was generally intuitive, misunderstandings still occurred, particularly among infrequent app users. Furthermore, the app was only available in German and was limited to bedside terminals rather than mobile devices. This design choice, while appropriate for inpatient monitoring, impedes future scalability and patient autonomy in outpatient settings. Previous studies showed that smartphone compatibility significantly increases flexibility and patient engagement [23]. Transitioning to mobile platforms and incorporating multilingual support will therefore be critical for broad adoption of the EVD.

Notably, this study did not assess the long-term adherence or completeness of entries beyond the immediate postoperative phase. While Johnson et al. and Sussman et al. reported lower completion rates for EVDs over extended periods [24, 25], our 24-hour snapshot benefited from a controlled hospital setting and consistent reminders. Nonetheless, this short observation period may have positively skewed compliance metrics [16]. Longer-term studies are needed to evaluate sustained use and real-world adherence in in- and outpatient settings.

Patient feedback further supports the clinical potential of the EVD. More than half of the participants rated the EVD as helpful, and nearly three-quarters reported no or only minimal burden during its use. The positive reception of the graphical interface is particularly relevant, as visual feedback has been shown to enhance patient understanding and motivation [7, 26]. However, almost half of the participants evaluated the process of entering results within the app as neither helpful nor unhelpful, or as unhelpful. This suggests that certain elements of the digital workflow may not yet fully meet patients’ needs. Refinements such as a more intuitive input process or improved in-app guidance appear warranted.

Based on insights from the open ended questions, initial modifications—such as adjustments to button layout and the organization of support sections—have already been implemented. Future app versions could not only evaluate the new features but also explore the integration of avatars or gamification strategies, similar to digital coaching concepts described by Andrade et al., to further enhance engagement and support rehabilitation activities, including pelvic floor training [27].

The results of this feasibility study should be interpreted in the context of several limitations. First, the sample size was small and the study was conducted at a single center, which restricts the generalizability of our findings. Importantly, recruitment was necessarily limited to the urology “living lab” ward because the intervention relied on in-room screens. While this setting enabled a controlled evaluation of initial usability and workflow integration, it also meant that RARP patients treated on the other wards could not be approached or included, and an overall departmental enrollment rate could not be reported. Second, selection bias cannot be excluded: patients who feel more comfortable with digital technologies may have been more likely to participate, which may have inflated usability ratings and task success. Finally, although socioeconomic and educational factors are increasingly recognized as important determinants of technology acceptance, our cohort was not large enough to allow meaningful subgroup analyses. Taken together, these limitations underscore that the present work is best viewed as an early, pragmatic step to assess feasibility and identify barriers, and they highlight the need for future studies with broader recruitment strategies and larger, more diverse patient populations.

Given the small sample size, we were unable to perform meaningful subgroup analyses (e.g., by socioeconomic or educational background), which may influence usability outcomes and should be addressed in larger future studies.

Despite these limitations, the present study was intentionally designed as a feasibility study. In this context, the findings provide relevant preliminary data regarding the practicality and acceptability of the intervention and may serve as a basis for future multicenter studies with larger and more diverse patient populations.

The continued evolution of eHealth infrastructure and increasing digital literacy among patients provide a favourable context for broader implementation of tools like the EVD. When embedded into existing hospital IT systems, these tools can facilitate continuous, structured monitoring while reducing the burden on both patients and healthcare professionals. As Vaccari et al. and Quinn et al. have pointed out, such innovations not only improve compliance but also contribute to a more patient-centered, data-driven model of care [7, 19].

## Conclusion

This exploratory feasibility study demonstrates that the EVD is a practical, reliable, and well-accepted alternative to the PVD in the early postoperative phase after RARP. With a high overall task success rate and favorable SUS scores, particularly among younger, digitally experienced, and regular app users, the EVD proved both efficient and user-friendly. Compared directly to the PVD as the current gold standard, it was rated superior in ease of use and graphical presentation, though misunderstandings occurred more often in older or infrequent app users. Beyond the frequency of such misunderstandings, future research should also assess their clinical relevance. Despite the small sample size, these findings provide a strong basis for broader implementation of EVDs in urological care, where they could play a key role in in enhancing patient engagement, optimizing clinical workflows, and supporting timely, personalized postoperative decision-making.

## Supplementary Information

Below is the link to the electronic supplementary material.

Supplementary Material 1

## Data Availability

The data that support the findings of this study are not openly available due to reasons of sensitivity and are available from the corresponding author upon reasonable request.

## Abbreviations

EVD: Electronic voiding diary
PVD: Paper-based voiding diary
RARP: Robot-assisted radical prostatectomy
SUS: System usability scale
