# Supplementary material for: Digital versus paper-based voiding diaries: a feasibility study of usability, data quality, patient satisfaction and adherence
Source: World J Urol. 2026 Jul 20;44(1):507. doi: 10.1007/s00345-026-06609-5 (PMC13385126; doi:10.1007/s00345-026-06609-5)
Supplement: Supplementary file 1 — Supplementary Material 1 [file 345_2026_6609_MOESM1_ESM.pdf]

Evaluation eines digitalen, App-basierten Miktionsprotokolls bei Patientinnen und Patienten nach urologischer Operation

1. **Wie alt sind Sie?**

____________________________ (Alter in Jahren)

2. **Sind Sie**
   - ◻ Männlich
   - ◻ Weiblich
   - ◻ Divers

3. **Haben Sie in der Vergangenheit schonmal ein Miktionsprotokoll ausgefüllt?**
   - ◻ Ja
   - ◻ Nein

4. **Sind Sie**
   - ◻ Alleinstehend
   - ◻ Verheiratet
   - ◻ Geschieden
   - ◻ Verwitwet

5. **Höchster Ausbildungsstatus**
   - ◻ Hauptschule
   - ◻ Realschule
   - ◻ Gymnasium
   - ◻ Universität
   - ◻ Andere ____________________________

6. **Benutzen Sie regelmäßig Apps?**
   - ◻ Ja
   - ◻ Nein

7. **Haben Sie schonmal eine Gesundheits-bezogene App (eine App, die mit Gesundheitsdaten arbeitet, ähnlich wie das digitale Miktionsprotokoll) benutzt?**
   - ◻ Ja
   - ◻ Nein

8. **Wie viele Stunden pro Tag benutzen Sie ihr Handy oder Tablet?**

   _______________________ Stunden

9. Wie lange ist Ihr letzter Krankenhausaufenthalt her?

- □ < 1 Jahr
- □ 1-5 Jahre
- □ > 10 Jahre
- □ Ich war vorher noch nie im Krankenhaus

10. Würden Sie die App einem Freund / einer Freundin weiterempfehlen?

- □ Ja, sehr
- □ Ja, etwas
- □ Weder noch
- □ Nein, eher nicht
- □ Nein, gar nicht

11. Haben Sie das Eintragen von Ereignissen in die App als hilfreich für die Kontrolle nach der Operation empfunden?

- □ Ja, sehr
- □ Ja, etwas
- □ Weder noch
- □ Nein, eher nicht
- □ Nein, gar nicht

12. Hat es Sie belastet, dass Sie Ereignisse in die App eintragen mussten?

- □ Ja, sehr
- □ Ja, etwas
- □ Weder noch
- □ Nein, eher nicht
- □ Nein, gar nicht

13. Wo hatten Sie Schwierigkeiten im Umgang mit der App?

14. Was fanden Sie an der App besonders gut?

15. Haben Sie Verbesserungsvorschläge für die App?

Fragebogen zur System-Gebrauchstauglichkeit (SUS)

1. Ich denke, dass ich das digitale Miktionsprotokoll gerne häufig benutzen würde.

**Stimme überhaupt nicht zu<br>1**	**2**	**3**	**4**	**Stimme voll zu<br>5**
○	○	○	○	○

2. Ich fand das digitale Miktionsprotokoll unnötig komplex.

**Stimme überhaupt nicht zu<br>1**	**2**	**3**	**4**	**Stimme voll zu<br>5**
○	○	○	○	○

3. Ich fand das digitale Miktionsprotokoll einfach zu benutzen.

**Stimme überhaupt nicht zu<br>1**	**2**	**3**	**4**	**Stimme voll zu<br>5**
○	○	○	○	○

4. Ich glaube, ich würde die Hilfe einer technisch versierten Person benötigen, um das digitale Miktionsprotokoll benutzen zu können.

**Stimme überhaupt nicht zu<br>1**	**2**	**3**	**4**	**Stimme voll zu<br>5**
○	○	○	○	○

5. Ich fand, die verschiedenen Funktionen in diesem digitalen Miktionsprotokoll waren gut integriert.

**Stimme überhaupt nicht zu<br>1**	**2**	**3**	**4**	**Stimme voll zu<br>5**
○	○	○	○	○

6. Ich denke, das digitale Miktionsprotokoll enthielt zu viele Inkonsistenzen.

**Stimme überhaupt nicht zu<br>1**	**2**	**3**	**4**	**Stimme voll zu<br>5**
○	○	○	○	○

7. Ich kann mir vorstellen, dass die meisten Menschen den Umgang mit dem digitalen Miktionsprotokoll sehr schnell lernen.

**Stimme überhaupt nicht zu 1**	**2**	**3**	**4**	**Stimme voll zu 5**
◌	◌	◌	◌	◌

8. Ich fand das digitale Miktionsprotokoll sehr umständlich zu nutzen.

**Stimme überhaupt nicht zu 1**	**2**	**3**	**4**	**Stimme voll zu 5**
◌	◌	◌	◌	◌

9. Ich fühlte mich bei der Benutzung des digitalen Miktionsprotokolls sehr sicher.

**Stimme überhaupt nicht zu 1**	**2**	**3**	**4**	**Stimme voll zu 5**
◌	◌	◌	◌	◌

10. Ich musste eine Menge lernen, bevor ich anfangen konnte das digitale Miktionsprotokoll zu verwenden.

**Stimme überhaupt nicht zu 1**	**2**	**3**	**4**	**Stimme voll zu 5**
◌	◌	◌	◌	◌

Vergleich digitales und analoges (Papierform) Miktionsprotokoll

1. **Fanden Sie es einfacher, das digitale Miktionsprotokoll oder das schriftliche Miktionsprotokoll zu benutzen? Bitte kreuzen Sie in der unteren Spalte an.**

Digitales Miktionsprotokoll besser — Beide gleich gut — Schriftliches Miktionsprotokoll besser

4	3	2	1	0	1	2	3	4

2. **Für welches der beiden Protokolle trifft die folgende Aussage besser zu? „Ich finde, das Protokoll enthält zu viele Unverständlichkeiten.“ Bitte kreuzen Sie in der unteren Spalte an.**

Digitales Miktionsprotokoll — Keines davon — Schriftliches Miktionsprotokoll

4	3	2	1	0	1	2	3	4

3. **Der Umgang mit welchem der beiden Protokolle ist Ihrer Meinung nach leichter zu erlernen? Bitte kreuzen Sie in der Spalte an.**

Digitales Miktionsprotokoll — Keines davon — Schriftliches Miktionsprotokoll

4	3	2	1	0	1	2	3	4

4. **Mit welchem Protokoll lassen sich die Ergebnisse aus Ihrer Sicht besser erfassen (darstellen)? Bitte kreuzen Sie in der Spalte an.**

Digitales Miktionsprotokoll besser — Beide gleich gut — Schriftliches Miktionsprotokoll besser

4	3	2	1	0	1	2	3	4
